# Absolute Lymphocyte Count as a Predictor of Mortality in Emergency Department Patients with Paraquat Poisoning

**DOI:** 10.1371/journal.pone.0078160

**Published:** 2013-10-24

**Authors:** Changwoo Kang, Seong Chun Kim, Soo Hoon Lee, Jin Hee Jeong, Dong Seob Kim, Dong Hoon Kim

**Affiliations:** Department of Emergency Medicine, Gyeongsang National University Hospital, Jinju, Korea; Kaohsiung Chang Gung Memorial Hospital, Taiwan

## Abstract

**Background:**

Paraquat (PQ) is a potent, highly toxic and widely used herbicide. The major medical problems associated with PQ are accidental or suicidal ingestion. There are several prognostic markers of PQ poisoning, with the serum PQ concentration considered to be the best indicator of outcome. However, the measurement of such markers is limited in many hospitals.

**Objective:**

The present study was conducted to investigate the association of absolute lymphocyte count (ALC) and the 30-day mortality rate in patients with PQ poisoning.

**Methods:**

We performed a retrospective analysis of patients admitted to the emergency department after paraquat poisoning between January 2010 and April 2013. Independent risk factors including ALC for 30-day mortality were determined. The ALC was categorized in quartiles as ≤1700, 1700 to 3200, 3200 to 5000, and >5000. Univariate and multivariate Cox proportional hazard analysis were performed to determine the independent risk factors for mortality.

**Results:**

A total of 136 patients were included in the study, and the 30-day mortality was 73.5%. ALC was significantly higher in nonsurvivors than in survivors. The highest ALC quartile (ALC>5000; hazard ratio, 2.58; 95% CI, 1.08–6.21) was associated with increased mortality in multivariate analysis. In addition, old age, lower arterial PaCO_2_, increased peripheral neutrophil count, and high serum levels of creatinine were associated with mortality.

**Conclusion:**

The absolute lymphocyte count is associated with the 30-day mortality rate in patients with paraquat poisoning.

## Introduction

Paraquat (1,1-dimethyl-4,4′-bipyridynium chloride; PQ) is one of the most widely used potent herbicides in the world. PQ is a non-selective, fast-acting contact herbicide. However, it is highly toxic when ingested. Accidental or suicidal ingestion are the major medical problems associated with PQ poisoning, with many fatalities reported [1]. The main toxic mechanism of PQ is a redox reaction by reactive oxygen species and lipid peroxidation of cellular membranes [2]. Moreover, it has been reported that inflammatory response is one mechanism of tissue injury after PQ poisoning.

Several prognostic markers and laboratory tests have been reported in the evaluation of patient severity, including plasma PQ concentration, arterial lactate, lactate metabolic clearance rate, modified Simplified Acute Physiology Score II (MSAPS II), modified SAPS IIe (MSAPS IIe), sequential organ failure assessment (SOFA), and modified SOFA (mSOFA) [1], [3]–[7]. Among the aforementioned predictors, the plasma PQ concentration is the most reliable marker for predicting death as a result of PQ poisoning [1].

The immune system can be affected by many chemicals, including pesticides [8]. PQ, one such pesticide, has been previously reported to cause increased immunomodulatory effects in vitro [9]. Absolute lymphocyte count (ALC) was found as an independent predictor of survival for patients with diffuse large B-cell lymphoma and follicular lymphomas [10]–[15]. Furthermore, low peripheral blood lymphocyte count was found to be associated with major adverse cardiovascular outcomes [16]–[20].

The association of ALC with clinical conditions associated with pesticide poisoning has not been evaluated. Thus, the aim of the present study was to investigate the potential role of the ALC as a prognostic marker of mortality in patients with PQ poisoning.

## Materials and Methods

### Ethics statement

This study complied with the statements of the Declaration of Helsinki and was approved by the institutional review board at the Gyeongsang National University Hospital with a waiver of informed consent.

### Study setting

We retrospectively analyzed patients admitted to a single emergency department (ED) with PQ poisoning between January 1, 2010 and April 31, 2013. A total of 136 patients were included in the study. Diagnosis of PQ poisoning was based on clinical history and a urine test. Patients were included in the present study if they were older than 18 years of age, suffered oral exposure to PQ, and had a positive urine test. Patients were excluded if PQ was combined with other drugs or if the patient was transferred to another hospital or discharged against medical advice. If the patient had critical underlying diseases such as malignancy, heart, lung, renal or liver diseases, the subject was excluded from the current study. Detoxification of PQ was conducted by gastric lavage with a large amount of saline followed by activated charcoal medication and charcoal hemoperfusion. Hemoperfusion was conducted after obtaining permission by the patient or the next of kin. A urine test for the determination of PQ poisoning was measured semi-quantitatively using a dithionite method.

### Data collection

Demographic parameters such as age and gender were collected. The time from ingestion to admittance to the ED and initial vital signs were also evaluated. The initial laboratory findings, including arterial pH, PaCO_2_, PaO_2_, white blood cell count, lymphocyte count, hematocrit, platelet count, plasma PQ concentration, and levels of blood urea nitrogen (BUN), creatinine, sodium, potassium, albumin, aspartate aminotransferase (AST), alanine aminotransferase (ALT) were also collected. The primary endpoint of this study was 30-day mortality after admission to the ED. However, if the patients were discharged within 30 days, we determined whether they participated in an outpatient department follow-up or were included in a telephone interview.

### Statistical analysis

The ALC was categorized by quartiles according to the number of patients: 1700 or less (n = 36), 1700 to 3200 (n = 35), 3200 to 5000 (n = 32), and 5000 or greater (n = 33). Continuous variables were examined as the mean and SD and were compared using a one-way analysis of variance. Male sex and 30-day mortality were described as the frequency of occurrence as a percentage and compared using a X^2^ test. The univariate and multivariate Cox regression analyses were presented as hazard ratios (HRs) with a 95% confidence interval. Variables that showed a P value less than 0.1 in the univariate analysis were included in the multivariate analysis. Survival curves were estimated using the Kaplan–Meier method and were compared using the log-rank test. The area under the receiver operating characteristic curve was used to discriminate the ALC with respect to 30-day mortality. P values less than 0.05 were considered to be significant. All of the analyses were performed using SPSS 21.0 software (SPSS Inc., IL).

## Results

In this retrospective cohort, a total of 200 cases were screened. Sixty-four cases were excluded, including 25 cases of non-intended oral exposure, 3 cases of ingestion combined with other drugs, 16 transfers to other hospitals, 14 cases of patient discharge against medical advice, and 6 cases of severe underlying diseases. Therefore, 136 patients were included in the analysis. The baseline demographics and clinical characteristics of the 136 patients are described in Table 1. In total, 91 (66.9%) men and 45 (33.1%) women were included, and the overall mean age was 60.2±16.6 years. One-hundred (73.5%) patients died within 30 days after ED admission due to PQ poisoning.

**Table 1:** Baseline characteristics of patients (n = 136).

Age (yr)	60.2±16.6
Male gender, n	91 (66.9)
Time from ingestion to ED (hr)	5.1±15.1
Initial vital signs	
MAP (mm Hg)	96.8±18.1
Heart rate (beats/min)	92.0±20.3
Respiratory rate (breaths/min)	22.4±5.4
Laboratory findings	
pH	7.3±0.1
PaCO_2_ (mm Hg)	28.1±9.7
PaO_2_ (mm Hg)	103.1±39.2
WBC (×10^3^/mm^3^)	16.1±9.1
Neutrophil (×10^3^/mm^3^)	11.4±8.4
Lymphocyte (×10^3^/mm^3^)	3.7±2.6
≤1.7	36 (26.5)
1.7–3.2	35 (25.7)
3.2–5.0	32 (23.5)
>5.0	33 (24.3)
Hematocrit (%)	41.7±5.8
Platelet (×10^3^/mm^3^)	299.3±97.1
BUN (mg/dL)	17.9±11.4
Creatinine (mg/dL)	1.7±1.4
Sodium (mmol/L)	139.2±3.7
Potassium (mmol/L)	3.0±0.7
Albumin (g/dL)	4.3±0.6
AST (U/L)	58.7±59.7
ALT (U/L)	33.0±46.4
30-day mortality	100 (73.5)

Data were presented as means with SDs or number with percentage. MAP, mean arterial pressure; PaCO_2_, partial pressure of carbon dioxide; PaO_2_, partial pressure of oxygen; BUN, blood urea nitrogen; AST, aspartate transaminase; ALT, alanine transaminase.

**Figure 1:**
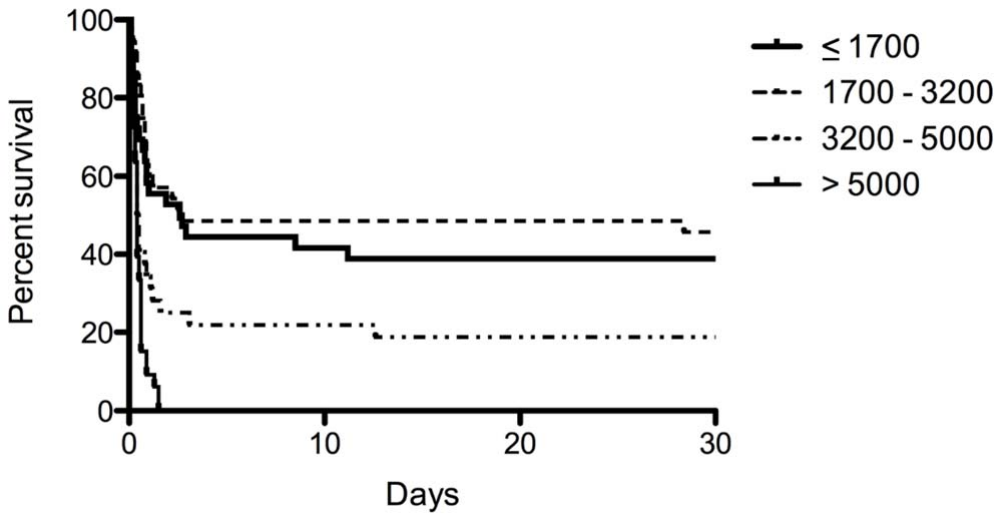
Kaplan-Meyer survival curve of patients with paraquat poisoning. P<0.001 by the log-rank test.

**Figure 2:**
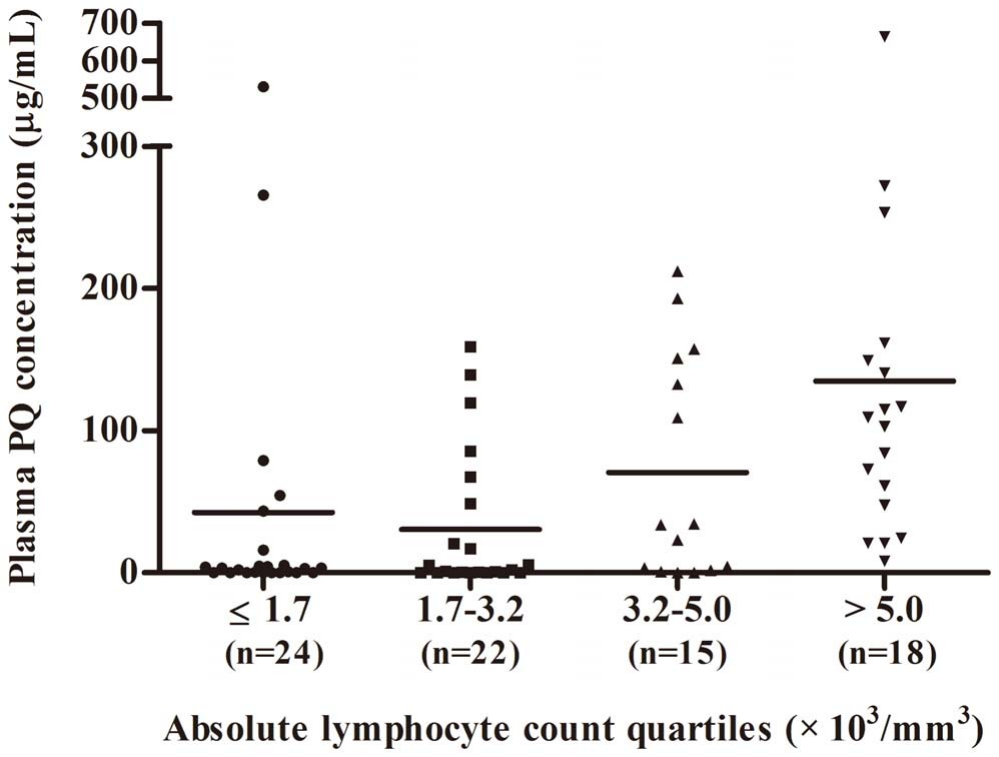
Scatter plot of plasma PQ concentration with line at mean in the absolute lymphocyte count quartiles.

Table 2 describes the patient characteristics of the study population stratified according to ALC levels upon ED admission. The patients in the highest ALC quartile (ALC>5000) were significantly older and had the lowest serum potassium levels. The time from ingestion to ED admission and initial laboratory findings including arterial pH, peripheral blood platelet count, serum creatinine, AST, ALT, and plasma PQ concentration were significantly different among stratified groups. Additionally, the mortality rate increased with an increase in the quartile of ALC. The mortality rates associated with the ALC group were 61.1% in ALC<1700, 54.3% in 1700≤ALC<3200, 81.2% in 3200≤ALC<5000, and 100% in ALC>5000 (P<0.001). A Kaplan-Meier curve was constructed using a log-rank test (Fig. 1). The results showed that increased ALC quartiles were associated with an increase in 30-day mortality. In addition, the ALC quartiles were compared to the plasma PQ concentration and the results are shown in Fig. 2.

**Table 2:** Patient characteristics stratified according to absolute lymphocyte count quartiles.

	≤1.7 (n = 36)	1.7–3.2 (n = 35)	3.2–5.0 (n = 32)	>5.0 (n = 33)	*p*
Age (yr)	51.7±18.1	59.3±13.7	64.7±15.1	66.2±15.6	0.001
Male gender, n	22 (61.1)	20 (57.1)	24 (75.0)	25 (75.8)	0.244
Time from ingestion to ED (hr)	11.6±28.1	3.3±3.9	2.3±3.2	2.7±3.2	0.028
Initial vital signs					
MAP (mm Hg)	100.7±17.3	99.2±16.0	93.8±19.8	93.0±18.8	0.200
Heart rate (beats/min)	88.9±17.9	90.5±22.9	90.2±17.4	98.9±21.9	0.161
Respiratory rate (breaths/min)	22.8±6.6	20.7±3.3	22.5±5.4	23.6±5.5	0.155
Laboratory findings					
pH	7.4±0.1	7.4±0.1	7.3±0.1	7.3±0.2	<0.001
PaCO_2_ (mm Hg)	28.2±8.8	30.1±10.7	27.3±9.6	26.6±9.4	0.526
PaO_2_ (mm Hg)	104.9±26.0	103.2±30.4	97.0±36.0	107.8±59.0	0.756
WBC (×10^3^/mm^3^)	15.7±9.3	14.2±8.8	15.9±9.4	18.7±8.6	0.234
Neutrophil (×10^3^/mm^3^)	13.6±9.3	11.0±8.4	10.7±9.0	10.2±7.6	0.350
Hematocrit (%)	41.9±6.7	40.5±4.7	41.9±6.6	42.6±5.0	0.488
Platelet (×10^3^/mm^3^)	247.0±60.6	316.9±100.3	289.0±92.5	347.7±104.1	<0.001
BUN (mg/dL)	21.6±19.4	16.9±7.6	15.9±5.5	16.9±5.0	0.150
Creatinine (mg/dL)	2.0±2.2	1.1±0.8	1.4±0.7	2.2±1.0	0.006
Sodium (mmol/L)	138.7±4.1	140.3±2.6	139.0±3.3	138.9±4.4	0.281
Potassium (mmol/L)	3.3±0.6	3.2±0.6	3.0±0.7	2.6±0.5	<0.001
Albumin (g/dL)	4.4±0.5	4.3±0.5	4.2±0.8	4.4±0.5	0.711
AST (U/L)	75.5±65.5	36.6±34.2	66.7±73.8	56.3±54.3	0.044
ALT (U/L)	52.5±77.6	22.1±21.1	29.8±30.1	26.7±23.7	0.030
Plasma PQ (µg/mL)*	42.6±118.0	30.6±50.4	70.4±79.1	134.7±151.5	0.015
30-day mortality	22 (61.1)	19 (54.3)	26 (81.2)	33 (100)	<0.001

Data were presented as means with SDs or number with percentage. MAP, mean arterial pressure; PaCO_2_, partial pressure of carbon dioxide; PaO_2_, partial pressure of oxygen; BUN, blood urea nitrogen; AST, aspartate transaminase; ALT, alanine transaminase.

* Plasma PQ concentration performed in 79 cases out of a total of 136 patients.

As shown in Table 3, the univariate analysis showed that age, heart rate, respiratory rate, pH, PaCO_2_, peripheral blood WBC, neutrophil count, platelet count, serum blood urea nitrogen, creatinine, potassium, AST, 3200 to 5000 ALC quartile, and highest ALC quartile group were significantly associated with 30-day mortality. In the multivariate Cox proportional hazards regression analyses, age, arterial PaCO_2_, peripheral blood neutrophil, serum creatinine, and highest ALC quartile (ALC>5000) were independent prognostic factors. Patients with an ALC of 5000 or greater had a 2.58-fold higher risk of death compared to patients with an ALC of 1700 or less during the 30-day follow-up period (Table 4). The area under the receiver operating characteristic curve for the ALC was 0.730 (95%CI, 0.646–0.814).

**Table 3:** Univariate analysis of risk factors for mortality within 30 days.

	Hazard ratio	95% confidence interval	*p*
Age (yr)	1.04	1.03–1.06	<0.001
Male gender, n	1.08	0.71–1.63	0.728
Time from ingestion to ED (hr)	1.01	0.99–1.01	0.709
Initial vital signs			
MAP (mm Hg)	1.01	0.99–1.02	0.397
Heart rate (beats/min)	1.02	1.00–1.03	0.009
Respiratory rate (breaths/min)	1.13	1.09–1.17	<0.001
Laboratory findings			
pH	0.02	0.01–0.10	<0.001
PaCO_2_ (mm Hg)	0.91	0.89–0.94	<0.001
PaO_2_ (mm Hg)	1.01	1.00–1.01	0.807
WBC (×10^3^/mm^3^)	1.07	1.05–1.09	<0.001
Neutrophil (×10^3^/mm^3^)	1.06	1.03–1.08	<0.001
Hematocrit (%)	1.01	0.97–1.05	0.726
Platelet (×10^3^/mm^3^)	1.01	1.00–1.01	0.092
BUN (mg/dL)	1.01	1.00–1.02	0.077
Creatinine (mg/dL)	1.21	1.11–1.32	<0.001
Sodium (mmol/L)	0.96	0.91–1.02	0.170
Potassium (mmol/L)	0.40	0.29–0.56	<0.001
Albumin (g/dL)	1.10	0.73–1.64	0.662
AST (U/L)	1.01	1.00–1.01	0.008
ALT (U/L)	1.00	1.00–1.01	0.942
Lymphocyte count (×10^3^/mm^3^)			
≤1.7	Reference		
1.7–3.2	0.80	0.43–1.48	0.480
3.2–5.0	1.98	1.12–3.50	0.019
>5.0	3.84	2.17–6.79	<0.001

MAP, mean arterial pressure; PaCO_2_, partial pressure of carbon dioxide; PaO_2_, partial pressure of oxygen; BUN, blood urea nitrogen; AST, aspartate transaminase; ALT, alanine transaminase.

**Table 4:** Multivariable-adjusted hazard ratios for mortality within 30 days.

	Hazard ratio	95% confidence interval	*p*
Age (yr)	1.03	1.02–1.05	<0.001
Heart rate (beats/min)	1.00	0.98–1.01	0.725
Respiratory rate (breaths/min)	1.05	0.99–1.10	0.099
pH	1.42	0.16–12.69	0.752
PaCO_2_ (mm Hg)	0.95	0.91–0.98	0.003
Neutrophil (×10^3^/mm^3^)	1.05	1.01–1.08	0.006
Platelet (×10^3^/mm^3^)	1.00	1.00–1.01	0.497
BUN (mg/dL)	0.98	0.95–1.01	0.277
Creatinine (mg/dL)	1.33	1.06–1.66	0.013
Potassium (mmol/L)	0.81	0.52–1.28	0.365
AST (U/L)	1.00	1.00–1.01	0.571
lymphocyte count (×10^3^/mm^3^)			
≤1.7	Reference		
1.7–3.2	0.76	0.36–1.61	0.474
3.2–5.0	1.52	0.69–3.37	0.302
>5.0	2.58	1.08–6.21	0.034

PaCO_2_, partial pressure of carbon dioxide; BUN, blood urea nitrogen; AST, aspartate transaminase.

## Discussion

Intracellular PQ undergoes redox cycling, a process of alternate reduction and re-oxidation [2]. Superoxide radicals, which are highly reactive oxygen species, are generated during this process and can cause subsequent deleterious effects and direct cellular damage [21]. Among organs, PQ poisoning has the strongest effect on the lungs because PQ preferentially accumulates in the alveolar epithelium [2]. PQ poisoning shows a high mortality rate, ranging from 50% to 90%, and an effective treatment has not been developed [22].

A number of prognostic factors have been proposed to predict the risk stratification of patients with acute PQ poisoning. The most reliable predictors of death are the plasma PQ concentration and the amount of PQ consumed [1], [23]. However, serum PQ concentration assays are not available in most local hospitals, and the amount of ingested PQ is often difficult to estimate. The plasma PQ concentration cannot be determined in our hospital. Rather, the test must be outsourced, and the results are available several days later. For this reason, the test results cannot be used immediately, which is a significant problem in our emergency department.

Lymphocytes are a specific type of WBC, and they form an integral part of the immune system. Lymphocytes consist of three major types, including T cells, B cells, and natural killer (NK) cells. Their main function is to facilitate humoral and cellular immunity against infectious microorganisms and other foreign pathogens. The ALC is equal to the percentage of lymphocytes among the total number of WBCs. Lymphocytosis is defined as an ALC>4.0×10^9^/L, and lymphocytopenia is usually defined as an ALC<2.5×10^9^/L. Potential causes of lymphocytosis include viral and bacterial infections, trauma, cardiac arrest, post-splenectomy state, auto-immune diseases, other chronic diseases, and lymphoproliferative disorders, including chronic lymphocytic leukemia and lymphoma. Lymphocytopenia is often reversible and is commonly observed in the presence of pancytopenia. The most common causes of lymphocytopenia are associated with a range of disorders, such as bacterial or fungal sepsis, postoperative state, malignancy, use of corticosteroids, cytotoxic chemotherapy or radiation therapy, and trauma or hemorrhaging [24].

The association between ALC and mortality has been evaluated, and lower ALCs are considered a poor prognostic factor in malignancy patients, including those with diffuse large B-cell lymphoma and follicular lymphomas [10]–[15]. In addition, low ALCs combined with abnormal troponin-I levels are considered poor prognostic markers in predicting recurrent instability and death in patients with unstable angina pectoris [18]. A low relative lymphocyte count was also a prognostic indicator of mortality in patients with coronary artery disease, heart failure, and those who had received an implantable cardioverter defibrillator [16], [19], [20]. Based on these results, the ALC indicates host immune status and plays an important role as a surrogate marker of host immunity.

A number of chemical agents can affect one or more immune functions, resulting in either decreased immunocompetence (immunosuppression) or inappropriate immunostimulation [8], [25]. Immunosuppression may lead to repeated, prolonged, or more severe infections, as well as the progression of malignancy. Immunostimulation may result in immune-mediated diseases including hypersensitivity reactions and autoimmune diseases. In an experimental study on the immunostimulatory effects of PQ, Paolillo et al. reported that PQ activated the expression of genes involved in inflammation (CXL10, CXL11, and IL-10) in immortalized human HaCat keratinocytes [9].

The present study revealed that ALC displayed a graded association with 30-day mortality and was an independent prognostic factor in patients with PQ poisoning. Unlike previous studies, higher ALCs were related to poor 30-day prognoses in patients with PQ poisoning. The mechanism of the association between higher ALCs and 30-day mortality in patients with PQ poisoning is not clear. However, immunotoxic agents can induce both immunosuppression and immunostimulation, as mentioned above. Based on these facts, we assume that PQ poisoning induced immunostimulation and increased the ALC. One important finding of the present study was the strong prognostic value of the ALC to predict 30-day mortality after PQ poisoning. To the best of our knowledge, the present study is the first analysis of the association of ALC with outcomes of PQ poisoning. Our study can be used to predict outcomes of patients with PQ poisoning who were admitted to the ED.

The current study presented several limitations. First, the study was conducted in a single institution, and the results of the analysis may not be generally applicable. Second, the present investigation was a retrospective and observational study. Thus, prospective studies should be performed to confirm our findings. Third, the mechanism of the association between ALC and patient outcome is unclear. Further studies should be conducted to investigate exact mechanisms. Fourth, the usefulness of the ALC as an outcome predictor is weak due to the overall high mortality rate (73.5%). However, as mentioned earlier, PQ is a potent and highly toxic herbicide. In addition, PQ poisoning has been shown to have a high mortality rate, ranging from 50% to 90%.

## Conclusions

Based on the results of the present study, we concluded that relatively high initial levels of ALC were associated with 30-day mortality in patients with PQ poisoning. Therefore, the ALC could be used as a prognostic marker in PQ poisoning.
